# Bowel wall thickening at CT: simplifying the diagnosis

**DOI:** 10.1007/s13244-013-0308-y

**Published:** 2014-01-10

**Authors:** Teresa Fernandes, Maria I. Oliveira, Ricardo Castro, Bruno Araújo, Bárbara Viamonte, Rui Cunha

**Affiliations:** Department of Radiology, Hospital de São João, Alameda Prof. Hernâni Monteiro, 4200-319 Porto, Portugal

**Keywords:** Computed tomography, Inflammatory bowel disease, Small bowel intestinal neoplasms

## Abstract

**Objective:**

In this article we present a simplified algorithm-based approach to the thickening of the small and large bowel wall detected on routine computed tomography (CT) of the abdomen.

**Background:**

Thickening of the small or large bowel wall may be caused by neoplastic, inflammatory, infectious, or ischaemic conditions. First, distinction should be made between focal and segmental or diffuse wall thickening. In cases of focal thickening further analysis of the wall symmetry and perienteric anomalies allows distinguishing between neoplasms and inflammatory conditions. In cases of segmental or diffuse thickening, the pattern of attenuation in light of clinical findings helps narrowing the differential diagnosis.

**Conclusion:**

Focal bowel wall thickening may be caused by tumours or inflammatory conditions. Bowel tumours may appear as either regular and symmetric or irregular or asymmetric thickening. When fat stranding is disproportionately more severe than the degree of wall thickening, inflammatory conditions are more likely. With the exception of lymphoma, segmental or diffuse wall thickening is usually caused by benign conditions, such as ischaemic, infectious and inflammatory diseases.

**Key points:**

• *Thickening of the bowel wall may be focal (<5 cm) and segmental or diffuse (6-40 cm or >40 cm) in extension.*

• *Focal, irregular and asymmetrical thickening of the bowel wall suggests a malignancy.*

• *Perienteric fat stranding disproportionally more severe than the degree of wall thickening suggests an inflammatory condition.*

• *Regular, symmetric and homogeneous wall thickening is more frequently due to benign conditions, but can also be caused by neoplasms such as well-differentiated adenocarcinoma and lymphoma.*

• *Segmental or diffuse bowel wall thickening is usually caused by ischaemic, inflammatory or infectious conditions and the attenuation pattern is helpful in narrowing the differential diagnosis.*

## Introduction

With the development of multidetector computed tomography scanners (MDCT), computed tomography became an important tool in the detection and characterisation of bowel abnormalities. This technology makes possible the acquisition of isotropic data and affords the capability of performing high-resolution multiplanar reconstructions [1–6]. In particular, CT enterography acquired after luminal distention through the administration of high volumes of neutral contrast material (,1500-2,000 ml of water, water-methylcellulose solution, polyethylene glycol electrolyte solution or low-concentration barium) is helpful in displaying the thickness and mural enhancement of the small bowel wall [2]. Adequate preparation and distention of the bowel lumen is, however, not always possible in the acute setting. In addition, wall abnormalities of the small and large bowel may be incidentally detected in asymptomatic patients or in patients with nonspecific complaints. For these, the CT imaging technique applied in a significant proportion of patients is a conventional one and radiologists should have a high level of suspicion in the detection and interpretation of bowel wall abnormalities.

### Normal bowel wall

Acceptable bowel wall thickness values on CT strongly depend on the degree of bowel distension and vary widely in the literature. Some agreement, however, exists that the small bowel wall should not exceed 3 mm despite luminal distention, and the colonic wall can vary from 1 to 2 mm when the lumen is well distended to 5 mm when the wall is contracted or the lumen is collapsed [2–9].

The bowel wall normally enhances after the administration of intravenous contrast material. The mucosa is the most intensely enhancing layer of the bowel wall and when enhanced may appear as a distinct layer. In contrast, the submucosa is less vascularised and is seldom seen as a separate structure on CT scans unless it is oedematous, haemorrhagic or infiltrated by fat [10].

### Thickening of the bowel wall

Thickening of the bowel wall may be caused by several pathologic conditions or be a normal variant [4]. When thickening of the bowel wall is identified on CT, several imaging features must be assessed in order to narrow the differential diagnosis: length of involvement, degree of thickening, symmetric versus asymmetric involvement, pattern of attenuation and perienteric abnormalities [3, 4, 6]. Each of these features may have a different significance according to the acute or chronic onset of clinical symptoms and will be further discussed in an algorithm approach [6].

### Approach to the thickened bowel wall

**Fig. 1:**
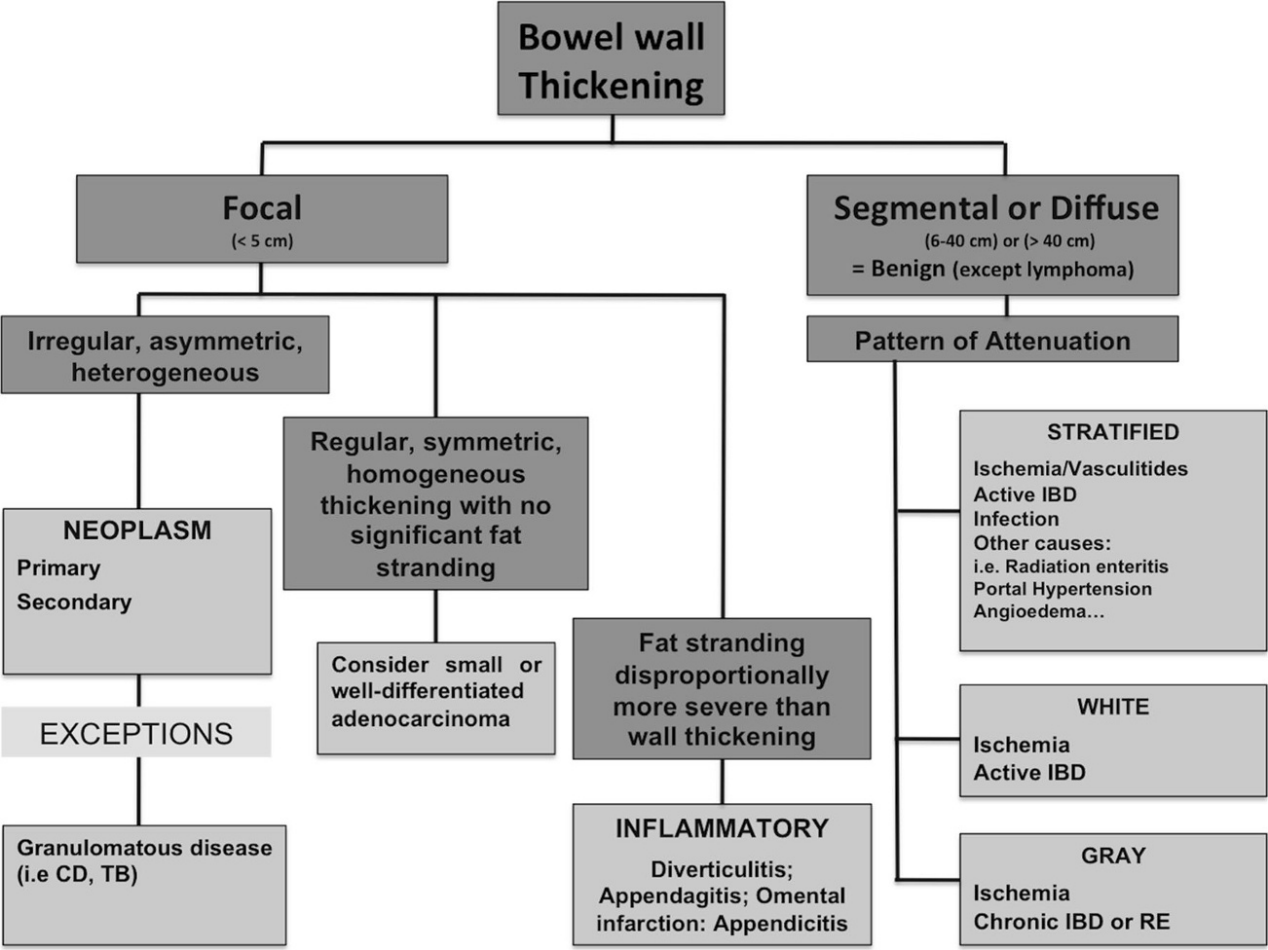
Algorithm approach to the bowel wall thickening. *CD* Crohn’s disease, *TB* tuberculosis, *IBD* inflammatory bowel disease, *RE* radiation enteritis. *Adapted from the electronic poster “Bowel wall thickening—a complex subject made simple” DOI:*10.5444/esgar2011/EE-063

When thickening of the small or large bowel wall is identified on CT, the first step to take is to access the extent of the involved bowel. Distinction should be made between **(1)** focal (less than 5 cm of extension) and **(2)** segmental (6-40 cm) or diffuse (>40 cm) involvement [3]. This is an important step in differentiating between benign and malignant causes of bowel wall thickening: while most bowel tumours present as a focal involvement, segmental and diffuse thickening of the bowel wall are usually caused by benign conditions [10]. The exception is a small bowel lymphoma, which typically shows as a segmental distribution [3, 6] (Fig. 1).

## Focal thickening of the bowel wall

Thickening of the bowel wall is considered focal when it extends less than 5 cm [3, 11]. Focal thickening may be caused by tumours or by inflammatory conditions, and distinguishing between the two conditions should be attempted. In addition to the clinical presentation, analysis of the wall symmetry, degree of thickening and perienteric abnormalities provides additional information for the correct diagnosis. In the setting of focal wall thickening three main scenarios may occur: (1) asymmetric focal thickening, (2) symmetric focal thickening and (3) perienteric abnormalities (fat stranding) disproportionately greater than the degree of wall thickening.

### (1) Asymmetric focal thickening of the bowel wall

**Fig. 2:**
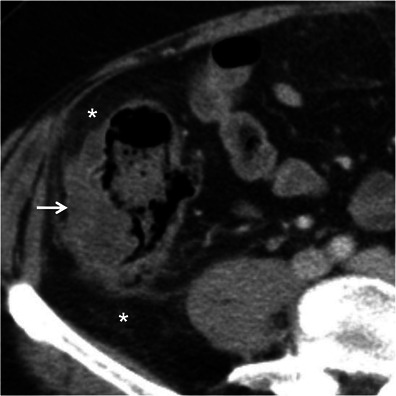
Colon cancer. Axial contrast-enhanced CT scan shows focal asymmetric and irregular thickening of the ascending colon (*arrow*), a finding strongly suggestive of a neoplasm. Also note mild pericolonic fat stranding (*asterisks*), a frequent associated finding. *Adapted from the electronic poster “Bowel wall thickening—a complex subject made simple” DOI:*10.5444/esgar2011/EE-063

Asymmetric thickening of the bowel wall corresponds to different degrees of eccentric thickening around the circumference of the involved segment and is typically caused by neoplasms [3, 12]. Malignant tumours of the gastrointestinal tract are more common in the stomach and colon and are less frequent in the small bowel, where they tend to occur at the proximal segments [11]. Neoplasms have a chronic onset and may present as an eccentric focal mass or, more commonly, as a circumferential asymmetric thickening, usually greater than 3 cm in thickness [3, 4, 10, 11, 13] (Fig. 2).

In this setting the attenuation pattern of the bowel wall after intravenous contrast administration and the perienteric abnormalities may be helpful in establishing the diagnosis. Contrast enhancement of malignant bowel tumours is frequently heterogeneous with areas of low attenuation due to ischaemia and necrosis [4, 10, 11]. This is particularly common on large and high-grade poorly differentiated tumours such as adenocarcinoma and stromal cell tumours [4]. In addition, regional adenopathy and distant metastases, when present, support the diagnosis [11].

#### Exceptions

Although asymmetric and heterogeneous focal thickening of the bowel wall usually indicates a malignancy, benign inflammatory conditions such as intestinal tuberculosis and Crohn’s disease may present with similar imaging features, sometimes mimicking neoplasms [3, 14, 15].

**Fig. 3:**
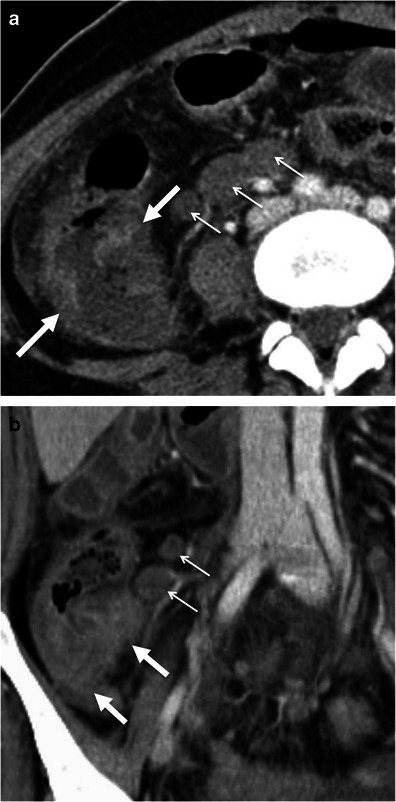
Intestinal tuberculosis. Axial (**a**) and reformatted coronal (**b**) contrast-enhanced CT scans show parietal irregular and asymmetric thickening of the caecum (*large arrows*), an appearance that mimics colon cancer. Also note low attenuation adenopathy (*thin arrows*), a usual finding in tuberculosis. Mild pericolonic fat stranding is also seen. *Adapted from the electronic poster “Bowel wall thickening—a complex subject made simple” DOI:*10.5444/esgar2011/EE-063

Gastrointestinal tuberculosis is rare. When present, however, it often involves the ileocaecal region. The inflammatory reaction usually produces eccentric wall thickening or a mass-like lesion. Discontinuous areas of mural thickening with associated luminal narrowing in the small bowel are also common and in combination with ileocaecal involvement should suggest the diagnosis. Large perienteric lymph nodes of low attenuation due to caseous necrosis are also common and characteristic (Fig. 3). These are not common in Crohn’s disease and would be unusual for caecal carcinoma [15, 16].

In addition, thoracic features of tuberculosis and other abdominal signs of involvement such as findings of peritonitis and hepatosplenic dissemination support the diagnosis.

**Fig. 4:**
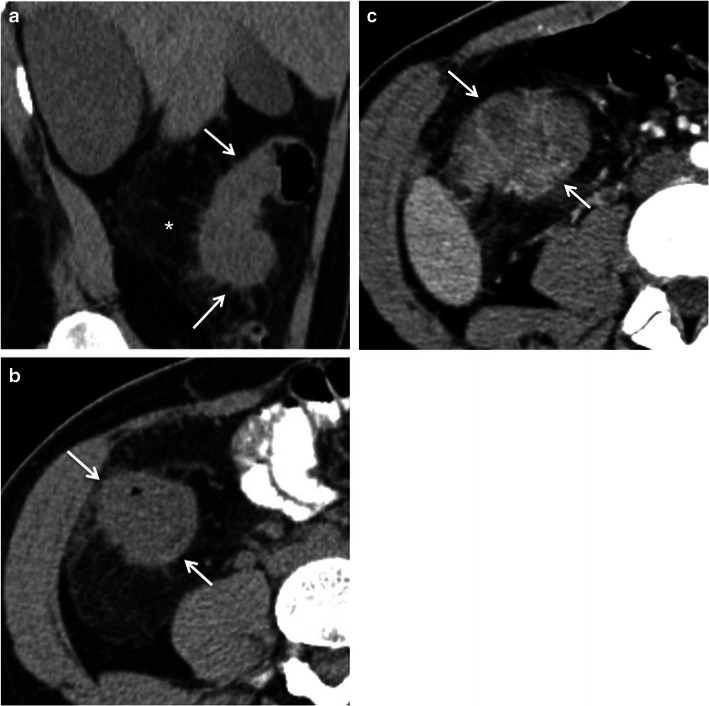
Crohn’s disease mimicking colon cancer. Reformatted coronal (**a**) and axial (**b**) unenhanced CT scans and axial contrast-enhanced CT scan (**c**) show spiculated irregular wall thickening of the caecal wall (*arrow* in **a**–**c**) with heterogeneous contrast enhancement (**c**). Also note proliferation of the pericaecal fat (*asterisk*), a common finding in Crohn’s disease. *Adapted from the electronic poster “Bowel wall thickening—a complex subject made simple” DOI:*10.5444/esgar2011/EE-063

Crohn’s disease typically involves the right colon and the terminal ileum. Wall thickening in Crohn’s disease is usually eccentric or asymmetric because of preferential involvement along the mesenteric border of the bowel wall [2, 7] (Fig. 4). Imaging features suggesting this diagnosis include the discontinuous involvement of the bowel wall (“skip areas”), signs of transmural inflammation such as fistulas and abscesses, and proliferation of the fat along the mesenteric border of the bowel [2, 3, 7].

### (2) Symmetric focal thickening of the bowel wall

**Fig. 5:**
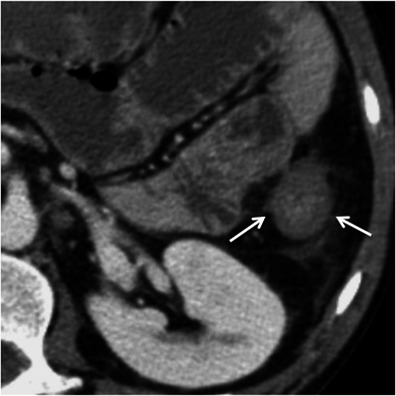
Well-differentiated adenocarcinoma of the descending colon. Axial contrast-enhanced CT scan showing focal concentric and regular thickening of the descending colon (*arrows*) with smooth contours and homogeneous enhancement. This proved to be a well-differentiated adenocarcinoma after biopsy. *Adapted from the electronic poster “Bowel wall thickening—a complex subject made simple” DOI:*10.5444/esgar2011/EE-063

Circumferential and symmetric thickenings of the bowel wall are features usually attributed to benign conditions such as inflammatory, infections, bowel oedema and ischaemia [3, 4]. However, neoplasms such as well-differentiated or small adenocarcinomas may also display symmetric and homogeneous thickening of the bowel wall and should be considered specially when the thickened bowel has a focal extension and no significant perienteric fat stranding is seen [4] (Fig. 5).

### (3) Perienteric abnormalities (fat stranding) disproportionately greater than the degree of bowel wall thickening

Inflammatory or infectious diseases of the bowel are usually centred in the bowel wall and can cause segmental or diffuse wall thickening [17]. However, in a few inflammatory enteric or perienteric conditions, the inflammatory changes are more prominent in the mesentery adjacent to the bowel rather than in the bowel wall itself. In these conditions, the bowel involvement is usually focal and mild, and the fat stranding is disproportionately greater than the degree of wall thickening. This is a helpful clue in narrowing the differential diagnosis to mainly four conditions: diverticulitis, epiploic appendagitis, omental infarction and appendicitis [17].

#### Diverticulitis

Diverticulae are sacculations of the mucosa and submucosa through the muscularis of the bowel wall, which are more common in the descending and the sigmoid colon. Diverticulitis occurs when the neck of a diverticulum becomes occluded, resulting in microperforation and pericolonic inflammation.

**Fig. 6:**
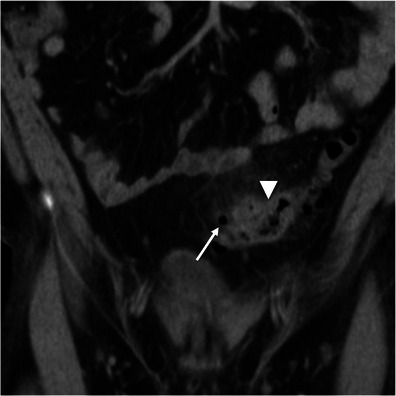
Acute diverticulitis. Reformatted coronal contrast-enhanced CT scan showing sigmoid diverticuli (*arrow*), mild bowel wall thickening (*arrowhead*) and pericolonic disproportionate fat stranding. These findings are compatible with sigmoid diverticulitis

CT findings of acute diverticulitis include inflamed diverticula in combination with pericolonic fat stranding, which is more severe than the mild focal thickening of the adjacent bowel wall [17]. Engorgement of the mesenteric vessels (“centipede” sign) and the presence of fluid at the base of the sigmoid mesentery (“comma sign”) are two indicative signs of the inflammatory process [17, 18] (Fig. 6).

Carcinoma of the colon is the most important differential diagnosis of diverticulitis when the wall thickening is more pronounced. The inflamed diverticula, homogeneous bowel wall enhancement, mesenteric signs of inflammation and lack of lymph nodes in light of the acute clinical presentation—localised pain and fever—support the diagnosis [18, 19].

#### Epiploic appendagitis

Epiploic appendages are pedunculated adipose structures protruding from the serosa surface of the colon into the peritoneal cavity. Acute epiploic appendagitis results from the torsion or venous occlusion of the epiploic appendage and is more frequent in the sigmoid colon [20].

**Fig. 7:**
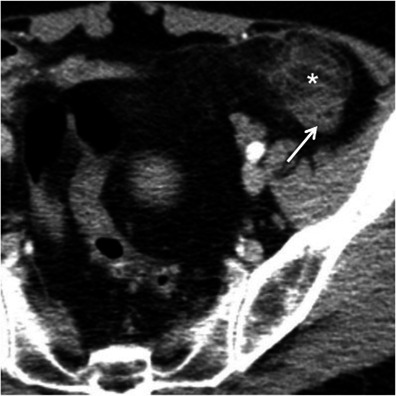
Epiploic appendagitis of the descending colon. Axial contrast-enhanced CT scan shows the inflamed epiploic appendage anterior to the colonic wall (*asterisk*) with adjacent disproportionate fat stranding and minimal wall thickening of the descending colon (*arrow*). *Adapted from the electronic poster “Bowel wall thickening—a complex subject made simple” DOI:*10.5444/esgar2011/EE-063

CT findings of epiploic appendagitis include the presence of a fat-density lesion corresponding to the inflamed appendix with surrounding inflammatory changes [20]. The engorged or thrombosed vessel may be seen as a high-attenuation focus within the fatty lesion (“central dot sign”), which constitutes a helpful finding to the diagnosis [20]. Mild reactive thickening of the colonic wall is often seen, but the paracolic inflammatory changes are disproportionately more severe [17, 20] (Fig. 7).

#### Omental infarction

**Fig. 8:**
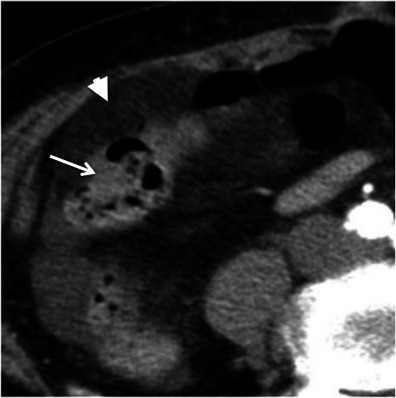
Omental infarction. Axial contrast-enhanced CT scan of a patient who presented with acute right upper quadrant pain shows an inhomogeneous fatty mass (*large arrow*) in the greater omentum, indicative of omental infarction. Note the mild wall thickening (*arrow*) of the adjacent colonic wall, which is clearly disproportionate relative to the fat stranding. *Adapted from the electronic poster “Bowel wall thickening—a complex subject made simple” DOI:*10.5444/esgar2011/EE-063

Infarction of the greater omentum may occur spontaneously, especially in obese people, or be secondary to abdominal surgery [7]. It is more common on the right side of the omentum and may clinically simulate appendicitis or cholecystitis. CT findings of omental infarction include a high-attenuation fatty mass centred in the omentum. Reactive bowel wall thickening of the colon may occur when the infarcted omentum is adjacent to it, but fat stranding is disproportionately more severe compared to the degree of bowel wall thickness [7] (Fig. 8).

#### Acute appendicitis

**Fig. 9:**
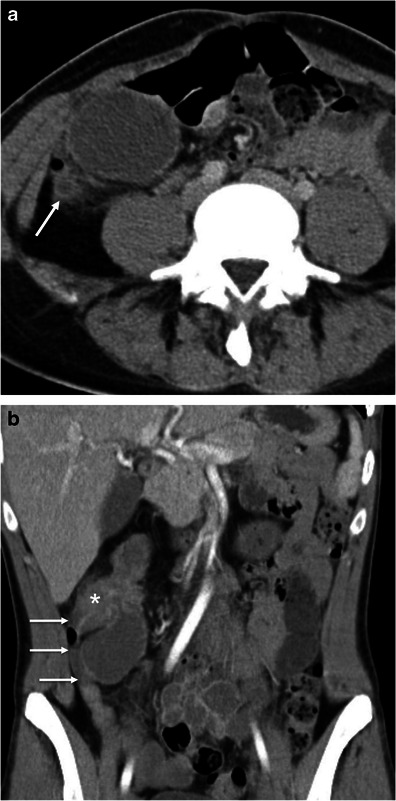
Acute appendicitis. Axial (**a**) and coronal (**b**) contrast-enhanced CT scan shows the retrocaecal enlarged fluid-filled appendix (*arrows*) associated with adjacent fat stranding and reactive wall thickening of the ascending colon (*asterisk*)

Acute appendicitis occurs when the appendiceal lumen becomes occluded, resulting in inflammation, ischaemia and eventually perforation [7, 17]. CT findings of acute appendicitis include a fluid-filled dilated (>6 mm in diameter) appendix, thickness of the wall, and mild to moderate peri-appendicular fat stranding. An appendicolith is present in up to 40 % of the cases. Mild thickening of the caecal apex wall may also occur (caecal bar and the arrowhead sign) [7, 17]. When the appendicitis is complicated with perforation and abscess formation, the appendix may be difficult to see. In these cases, severe fat stranding of the right lower quadrant is common and in the absence of substantial caecal and ileal thickening suggests the diagnosis [17] (Fig. 9).

## Segmental or diffuse bowel wall thickening

**Fig. 10:**
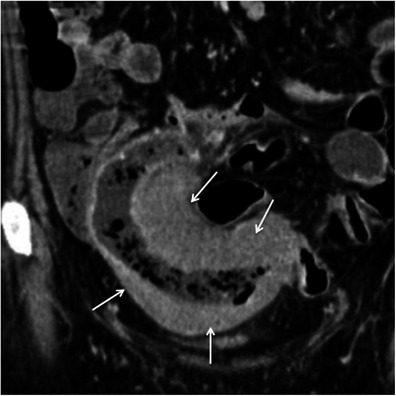
Small bowel lymphoma. Reformatted coronal contrast-enhanced CT scan shows concentric and regular segmental thickening of a small bowel loop (*arrows*) with homogeneous contrast enhancement. *Adapted from the electronic poster “Bowel wall thickening—a complex subject made simple” DOI:*10.5444/esgar2011/EE-063

When the thickened bowel has an extension of 6-40 cm or greater than 40 cm, it is considered a segmental or diffuse thickening respectively [3, 4]. Segmental or diffuse circumferential and symmetric thickening of the bowel wall is typically secondary to benign conditions and usually does not exceed 10 mm in thickness from the luminal to the serosal surface [10, 11]. As mentioned above, the exception is the small bowel lymphoma, which despite being a malignant condition may present with a segmental distribution causing circumferential symmetric thickening of the bowel wall and homogeneous low attenuation after intravenous contrast administration [3, 4, 10, 11] (Fig. 10).

In the setting of segmental or diffuse bowel wall thickening, one of three attenuation patterns after intravenous contrast administration may occur: a stratified attenuation pattern, white attenuation pattern or grey attenuation pattern [4, 6, 21].

## Stratified pattern of attenuation

In this pattern, two (double halo sign) or three (the target sign) concentric and symmetric layers of alternating densities are recognised on the thickened bowel wall after intravenous contrast administration.

This pattern indicates inflammation or ischaemia of the bowel where the inner and outer high-density layers correspond to the hyperemic mucosa and serosa, respectively, while the low-density layer presumably represents the oedematous submucosa [2–4, 6, 7, 11, 21].

Although generally indicative of benign conditions, these signs are not specific and may be present in several acute conditions. Clinical presentation and adjacent findings such as perienteric findings help in narrowing the differential diagnosis:

### Bowel ischaemia

Thickening of the bowel wall is the most common but least specific CT sign of bowel ischaemia [5, 22]. The extent of involvement, degree of thickness and pattern of attenuation of the ischaemic bowel vary according to three main factors: (1) pathogenesis of the ischaemia (arterial-occlusive, veno-occlusive or hypoperfusion); (2) severity of the ischaemia (transient ischaemia of the mucosa and/or submucosa versus transmural bowel wall necrosis); (3) superimposed haemorrhage or infection [5].

Although bowel wall thickening is a common finding in cases of bowel ischaemia, the ischaemic bowel wall may also appear paper thin, particularly in cases of acute arterial occlusion [5].

**Fig. 11:**
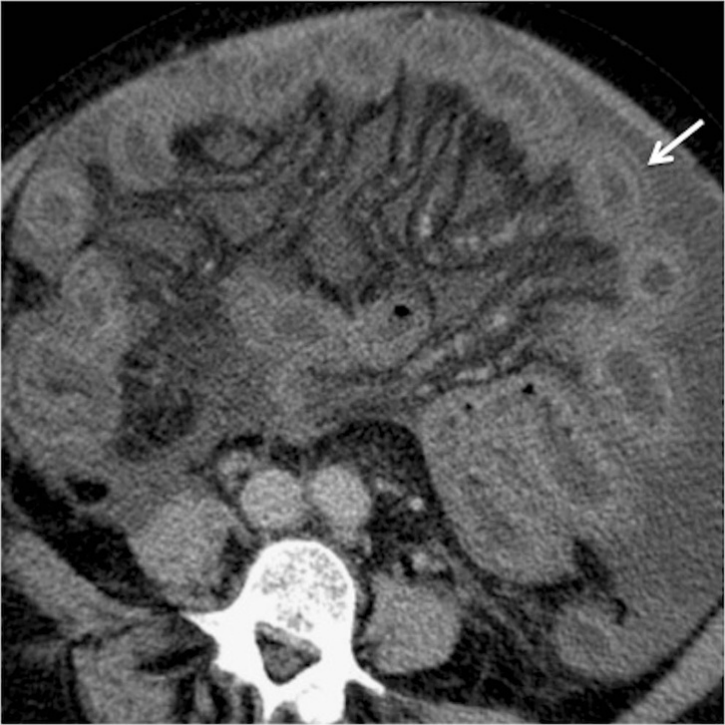
Acute small bowel ischaemia. Axial contrast-enhanced CT scan shows diffuse thickening of the small bowel loops with a target appearance (*arrow*) due to submucosal oedema. Also note the engorgement of the mesenteric root vessels and ascites, common findings in cases of acute bowel ischaemia. *Adapted from the electronic poster “Bowel wall thickening—a complex subject made simple” DOI:*10.5444/esgar2011/EE-063

When the ischaemic bowel wall is thickened, it may present with one or more of the three above-mentioned attenuation patterns referred [3, 5, 23]. The stratified pattern of attenuation may be an early finding of bowel ischaemia. This results from oedema of the submucosa and hyperaemia or hyperperfusion of the mucosa and/or muscularis propria [5, 6, 21, 24]. This finding should be judged in the clinical context and associated imaging findings of bowel ischaemia, such as occlusion of the mesenteric artery or vein, bowel dilatation, engorgement of the mesenteric veins, and mesenteric oedema and ascites [3, 11, 21, 22, 25] (Fig. 11). Intestinal pneumatosis and gas in the mesenteric or portal veins are indicative of severe ischaemia and are usually associated with the thinning rather than thickening of the small bowel wall due to bowel wall necrosis [24].

**Fig. 12:**
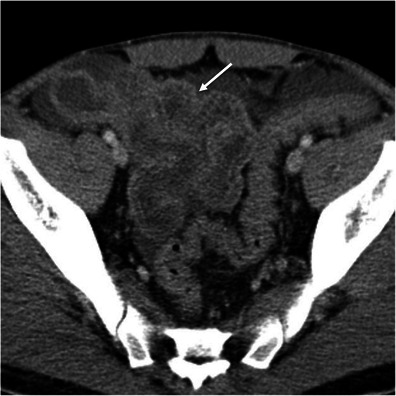
Bowel ischaemia secondary to systemic lupus erythaematosus (LES). Axial contrast-enhanced CT scan shows circumferential thickening of the small bowel loops (*arrows*) with a stratified appearance in a patient with LES presenting with bloody diarrhoea indicating bowel involvement by the vasculitis

Vasculitides are rare causes of gastrointestinal ischaemia with the highest prevalence in polyarteritis nodosa and usually present with thickening of the affected bowel wall with a stratified appearance [3, 24]. Distinguishing between ischaemia due to vasculitides and other causes of mesenteric ischaemia may be difficult based on radiologic findings alone (Fig. 12). This diagnosis, however, should be considered whenever mesenteric ischaemic changes occur in young patients; involve unusual sites such as the stomach, duodenum and rectum, and is not confined to a single vascular territory. In addition, systemic clinical manifestations (i.e. fever, weakness, malaise, myalgia and headache) point to the correct diagnosis [24].

### Idiopathic inflammatory bowel disease

Bowel wall thickening with a stratified pattern may be also seen in both ulcerative colitis (UC) and Crohn’s disease, indicating acute, active disease [2, 7, 26].

**Fig. 13:**
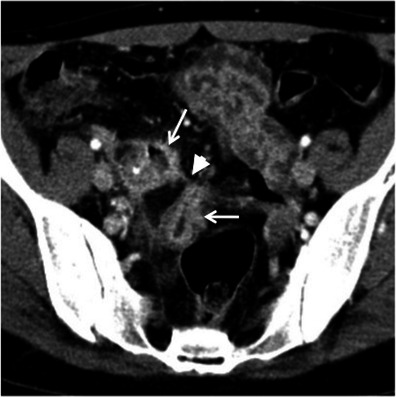
Stratified appearance in Crohn’s disease. Axial contrast-enhanced CT scan of the abdomen shows concentric wall thickening of small bowel loops with a stratified appearance indicating active disease (*arrows*). Also note a fistula (*arrowhead*) connecting the bowel loops, a common finding in Crohn’s disease

Crohn’s disease may occur in any part of the gastrointestinal tract but predominantly affects the small bowel, particularly the ileum and right colon [2, 7]. CT signs favouring Crohn’s disease include discontinuous involvement of the bowel wall (“skip areas”), prominent vasa recta (“comb sign”) and signs of transmural inflammation such as fistulas and abscesses, and proliferation of the fat along the mesenteric border of the bowel [2, 3, 7] (Fig. 13).

By contrast, UC is typically left sided, involves the rectum in 95 % of cases, and shows contiguous, circumferential and proximal extension through the colon [27]. The inflammatory process in UC is superficial, predominantly affecting the mucosa [7]. Thus, wall thickening and pericolonic involvement are not as extensive in ulcerative colitis as they are in Crohn’s disease [27].

### Infectious enteritis or colitis and pseudomembranous colitis

In most cases of infectious enteritis the small bowel wall appears normal or mildly thickened [3]. By contrast, infectious colitis typically manifests with significant wall thickening, which may demonstrate either homogeneous enhancement or a striated pattern due to intramural oedema. Stranding of the pericolic fat and ascites are also commonly seen [7, 28, 29]. Although the affected portion of the colon may suggest a specific organism, there is a considerable overlap of the appearances. Thus, laboratory studies are needed to achieve a definitive diagnosis [7].

**Fig. 14:**
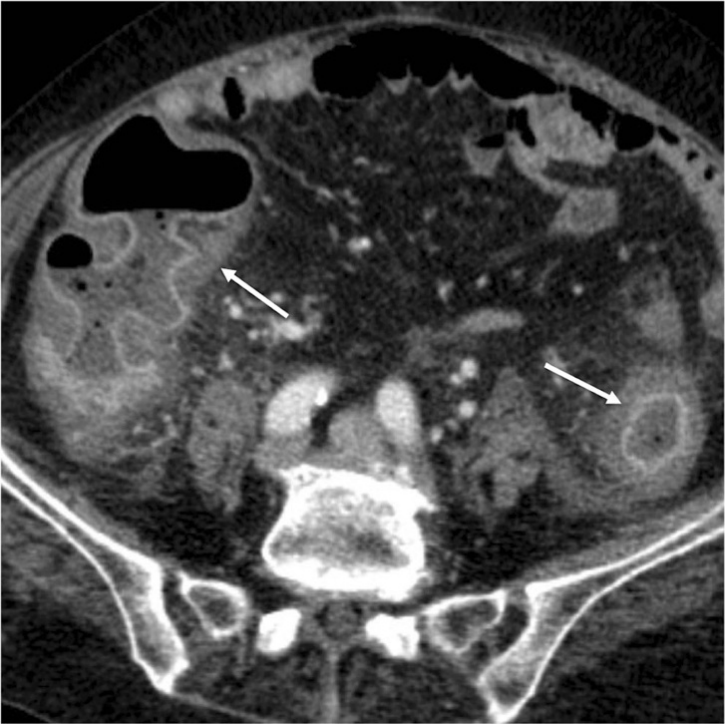
Stratified attenuation pattern in pseudomembranous colitis. Axial contrast-enhanced CT scan shows significant wall thickening of the ascending and descending colon (*arrows*) due to submucosal oedema, resulting in the stratified appearance. The marked thickening of the bowel wall and the mild pericolonic fat stranding suggest pseudomembranous colitis

Pseudomembranous colitis results from toxins produced by an overgrowth of the organism *Clostridium difficile* and usually presents as a pancolitis. The degree of bowel wall thickness in pseudomembranous colitis and cytomegalovirus colitis is usually greater than in any other inflammatory or infectious disease of the colon, while the pericolic fat stranding is often disproportionately mild [2, 9] (Fig. 14). After intravenous contrast administration, the thickened bowel wall may show low attenuation due to oedema, hyperenhancement due to hyperaemia or a striated appearance. When haustral folds are significantly thickened and protrude into the bowel lumen, they can trap the positive oral contrast material, an appearance known as the “accordion sign”. This sign suggests the diagnosis, although it may also occur from other causes of colitis [26].

### Specific clinical entities

**Fig. 15:**
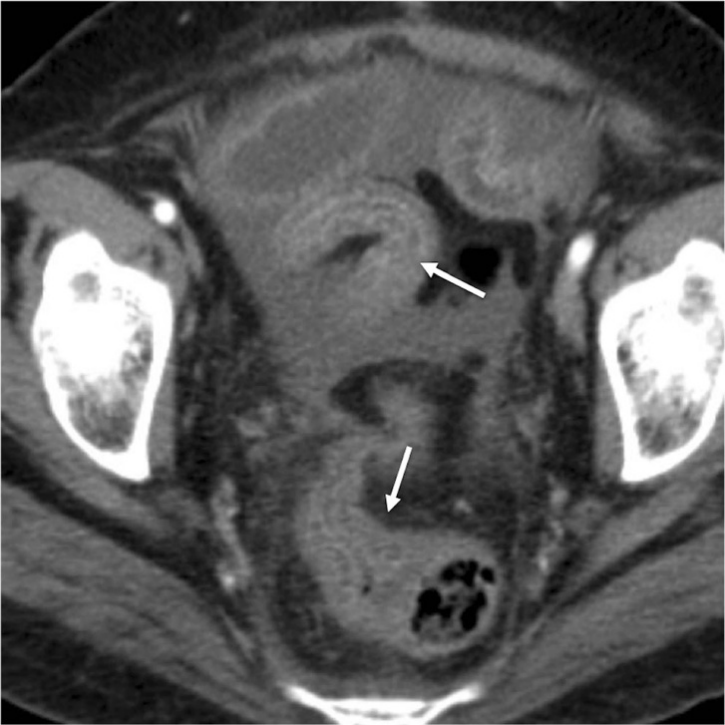
Radiation enteritis in a patient with cervical cancer. Axial contrast-enhanced CT scan shows concentric stratified wall thickening of low-lying small bowel loops (*arrows*). There are also some ascites. These findings in the context of radiation therapy are suggestive of radiation enteritis

**Fig. 16:**
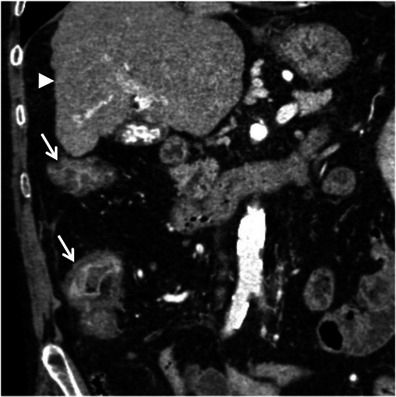
Stratified attenuation pattern in the ascending colon of a cirrhotic patient. Reformatted coronal contrast-enhanced CT scan shows parietal regular and concentric thickening of the ascending colon (*arrows*) with a “target” appearance due to oedema of the submucosa. Note the irregular contours of the liver (*arrowheads*) consistent with hepatic cirrhosis. *Adapted from the electronic poster “Bowel wall thickening—a complex subject made simple” DOI:*10.5444/esgar2011/EE-063

The striated attenuation may also be seen in specific clinical situations, such as graft-versus-host disease in patients submitted to allogeneic bone marrow transplantation, acute radiation enteritis or colitis in patients submitted to radiation therapy, bowel wall oedema in patients with a history of angioedema, and oedema of the right colon in cirrhotic patients [3, 7] (Figs. 15 and 16). In each of these conditions the appropriate clinical history is essential for establishing the correct diagnosis.

### Other causes of stratified appearance

Stratification of the bowel wall may also be caused by infiltration of the submucosa by tumour or fat. The rare infiltrating scirrhous carcinomas (linitis plastica) of the stomach or rectosigmoid may present with symmetric wall thickening, regular contours and stratification of the bowel wall [4, 11]. Narrowing of the intestinal lumen, regional adenopathy and distant metastasis point to the correct diagnosis [11].

**Fig. 17:**
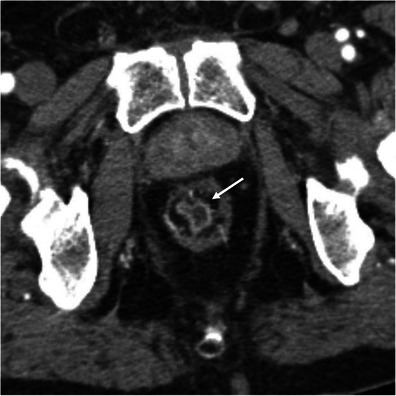
Fat halo sign in Crohn’s disease. Axial contrast-enhanced CT image shows concentric wall thickening of the rectum (*arrow*) with fatty appearance of the submucosa in a patient with a long history of Crohn’s disease

A target appearance may also be caused by deposition of fat in the submucosa, indicating past or chronic inflammation [2, 7]. It is more common in patients with idiopathic inflammatory bowel disease, especially ulcerative colitis (Fig. 17). Occasionally this sign can also be seen in patients with a history of radiation enteritis and even in patients with no history of gastrointestinal disease, where the intramural fat layer is usually much thinner than that seen in inflammatory bowel disease [3, 6, 27].

## White pattern of attenuation

The white pattern is caused by intense enhancement of the bowel wall when its density is equal to or greater than that of venous vessels in the same scan [6]. Visual assessment is usually sufficient to detect hyperenhancement of the wall when the bowel lumen is well distended [3]. This pattern can be seen mainly in two clinical entities: ischaemia and inflammatory bowel disease.

### Ischaemia

Hyperenhancement of the ischaemic bowel may occur because of the hyperaemia (i.e. mesenteric venous occlusion with outflow obstruction) or hyperperfusion (i.e. reperfusion after occlusive or nonocclusive ischaemia) of the bowel wall and is a good prognostic factor, indicating viability of the bowel wall [5, 22, 25]. As referred to above, associated imaging findings of bowel ischamia include occlusion of the mesenteric vessels, bowel dilatation, mesenteric oedema and ascites [3, 11, 22, 23, 26].

**Fig. 18:**
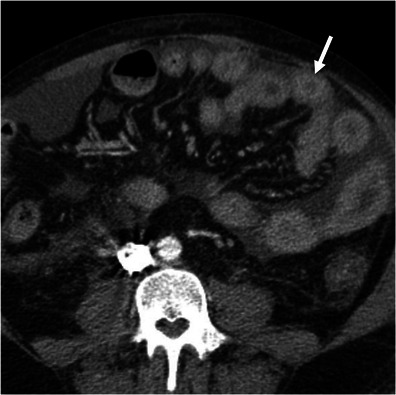
Shock bowel in a patient with significant haemorrhage due to bleeding oesophageal varices. Axial contrast-enhanced CT scan shows thickened hyperattenuating small bowel loops (*arrow*) due to the increased vascular permeability in the context of severe hypovolaemia. Also note engorgement of the mesenteric vessels and small volume ascites

The white pattern may also occur in patients with acute hypovolaemia or shock known as “shock bowel” [3] (Fig. 18). In this setting increased vascular permeability of the bowel wall leads to interstitial leakage of the contrast material resulting in higher attenuation [5, 6, 24, 30, 31].

**Fig. 19:**
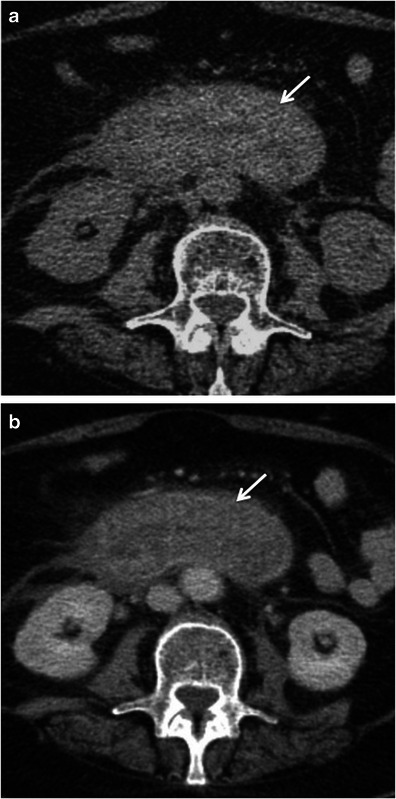
White attenuation pattern due to spontaneous intramural bowel wall haemorrhage in a patient with polyarteritis nodosa. Axial unenhanced (**a**) and contrast-enhanced (**b**) CT scans of the abdomen show concentric wall thickening of the third duodenal portion that showed spontaneous hyperenhancement of the bowel wall due to spontaneous haemorrhage. *Adapted from the electronic poster “Bowel wall thickening—a complex subject made simple” DOI:*10.5444/esgar2011/EE-063

Hyperattenuation of the bowel wall may also occur because of intramural haemorrhage in patients with bowel ischaemia, bleeding diathesis or undergoing anticoagulation therapy [5, 23]. Taking this into account, acquisition of both unenhanced and enhanced CT studies is essential in the distinction between hyperenhancement of the bowel wall and spontaneous hyperattenuation due to acute intramural haemorrhage [3, 4, 32] (Fig. 19).

### Inflammatory conditions

**Fig. 20:**
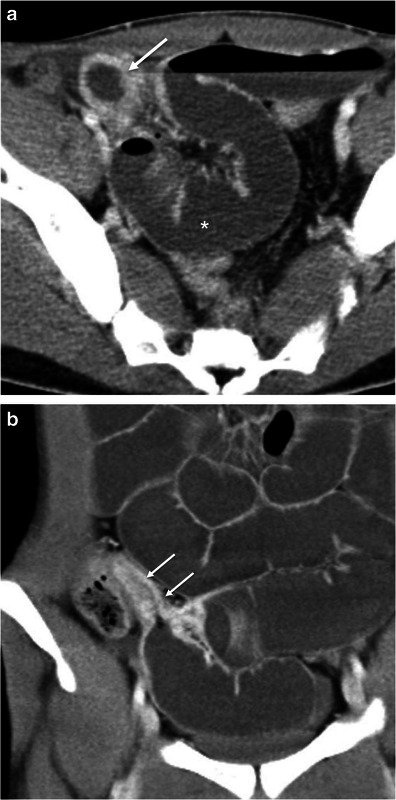
White attenuation pattern in Crohn’s disease. Axial (**a**) and coronal (**b**) contrast-enhanced CT scans show homogeneous hyperenhancement (*arrows*) of a thickened and stenotic ileal loop indicating active disease. Note the proximal dilatation of the small bowel loops (*asterisk*) due to the obstruction

Homogeneous hyperenhancement of the thickened bowel wall may also occur in the setting of infectious and idiopathic inflammatory bowel disease. In the latter case, hyperenhancement of the bowel wall indicates active disease [2, 3, 33] (Fig. 20).

## Grey pattern of attenuation

The grey pattern of attenuation indicates mild to diminished enhancement of the bowel wall and is considered when the attenuation of the bowel wall is similar to that of the muscle on contrast-enhanced scans [6]. In general this pattern corresponds to the least specific of the attenuation categories and so other imaging findings and clinical presentation are essential in establishing the correct diagnosis [6].

### Acute onset

**Fig. 21:**
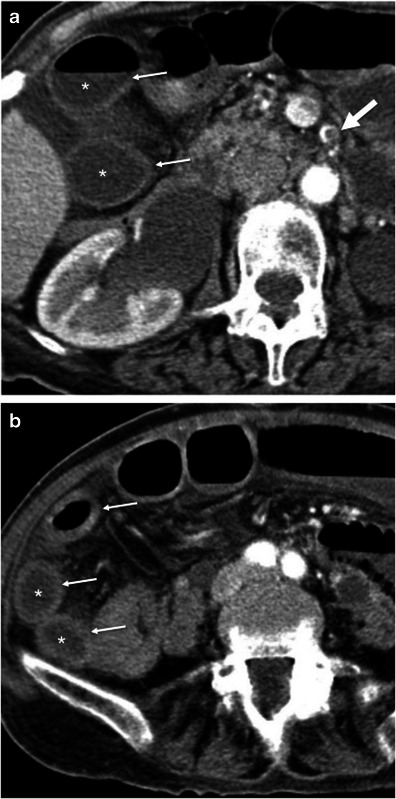
Grey attenuation pattern in small bowel ischaemia. Axial contrast-enhanced CT scans show distended fluid-filled small bowel loops (*asterisks* in **a** and **b**) with hypoenhancing thick walls (*thin arrows* in **a** and **b**) indicating ischaemia in a patient with partial occlusion of the superior mesenteric artery (*large arrow* in **a**). *Adapted from the electronic poster “Bowel wall thickening—a complex subject made simple” DOI:*10.5444/esgar2011/EE-063

**Fig. 22:**
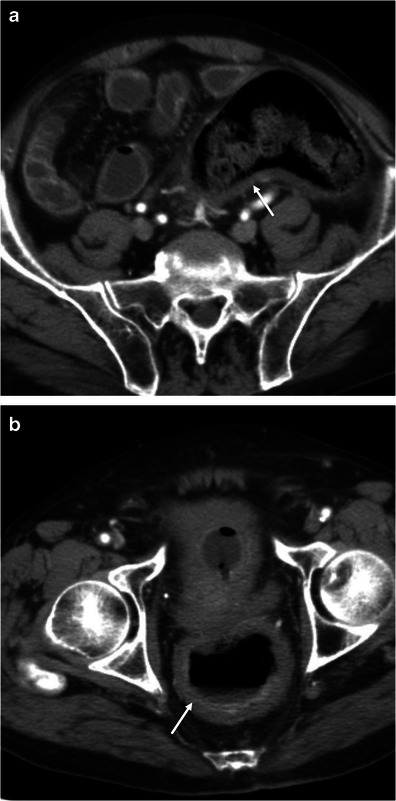
Ischaemic colitis. Axial contrast-enhanced CT scans through the descending colon (**a**) and rectum (**b**) show the thickened hypoattenuating bowel wall due to ischaemia. The diagnosis was confirmed with colonoscopy and biopsy

In the appropriate clinical setting decreased enhancement of the bowel wall may represent a compromise of the blood supply to the bowel wall and is pathognomonic of intestinal ischaemia [3, 10, 24]. The hypoattenuating bowel wall usually has a homogeneous appearance and is caused by bowel wall oedema [5] (Fig. 21). This pattern is particularly common in cases of mesenteric venous occlusion and bowel obstruction, where the bowel oedema is more pronounced due to venous congestion [23]. It is also frequent in ischaemic colitis, a common cause of abdominal pain in the elderly [7]. Ischaemic colitis results when blood flow to the colon is compromised, usually as a result of hypoperfusion. Although left-sided involvement is more common, diffuse or segmental involvement of the colon may occur depending on the cause and which vessels are involved [7] (Fig. 22).

### Delayed onset

In patients with chronic Crohn’s disease or chronic radiation enteritis, involved bowel loops may show diminished enhancement due to the development of transmural fibrosis [3, 4, 6]. Once transmural fibrosis has developed in Crohn’s disease, mural stratification is no longer seen. In this context, homogeneous low attenuation of the bowel indicates quiescence of the disease.

Chronic radiation enteritis or colitis may develop 6-24 months after completion of radiation therapy. Distribution is related to treatment fields and most frequently involves the rectum and sigmoid because of radiation for pelvic disease (most commonly prostatic or cervical cancer) [9]. CT findings of chronic radiation enteritis include hypoenhancing wall thickening, increased pelvic fat and thickening of the perienteric fibrous tissue [32]. Strictures and fistulas may also occur [33].

## Conclusion

Bowel wall thickening may be focal and segmental or diffuse. In cases of focal thickening, the degree and symmetry of thickening and perienteric abnormalities help narrow the differential diagnosis: while heterogeneous and asymmetric focal thickening is usually associated with malignancies, symmetric regular and homogeneous thickening may be caused by benign conditions but also well-differentiated tumours. Disproportionate fat stranding compared to the degree of wall thickening suggests inflammatory conditions. Segmental or diffuse bowel thickenings are usually caused by benign conditions, with the exception of lymphoma. Common causes include ischaemia, inflammatory and infectious conditions. The pattern of attenuation helps narrow the differential diagnosis of segmental or diffuse wall thickening but still there is a significant overlap on CT imaging findings of different non-neoplastic bowel conditions.

## References

[1] Fernandes TC, Castro R, Pinto D, Oliveira MI, Carneiro A, Cunha R (2011) Bowel wall thickening – a complex subject made simple. Electronic Poster presented at 2011 ESGAR meeting doi:10.5444/esgar2011/EE-063#_blank

[2] Paulsen SR, Huprich JE, Fletcher JG, Booya F, Young BM, Fidler JL, Johnson CD, Barlow JM, Earnest F (2006). CT enterography as a diagnostic tool in evaluating small bowel disorders: review of clinical experience with over 700 cases. Radiographics.

[3] Macari M, Megibow AJ, Balthazar EJ (2007). A pattern approach to the abnormal small bowel: observations at MDCT and CT enterography. AJR Am J Roentgenol.

[4] Macari M, Balthazar EJ (2001). CT of bowel wall thickening: significance and pitfalls of interpretation. AJR Am J Roentgenol.

[5] Wisner W, Khurana B, Ji H, Ros PR (2003). CT of acute bowel ischemia. Radiology.

[6] Wittenberg J, Harisinghani MG, Jhaveri K, Varghese J, Mueller PR (2002). Algorithmic approach to CT diagnosis of the abnormal bowel wall. Radiographics.

[7] Horton KM, Corl FM, Fishman EK (2000). CT evaluation of the colon: inflammatory disease. Radiographics.

[8] Fisher JK (1983). Abnormal colonic wall thickening on computed tomography. J Comput Assist Tomogr.

[9] Desai RK, Tagliabue JR, Wegryn SA, Einstein DM (1991). CT evaluation of wall thickening in the alimentary tract. Radiographics.

[10] Chou CK, Wu RH, Mak CW, Lin MP (2006). Clinical significance of poor CT enhancement of the thickened small-bowel wall in patients with acute abdominal pain. AJR Am J Roentgenol.

[11] Balthazar EJ (1991). CT of the gastrointestinal tract: principles and interpretation. AJR Am J Roentgenol.

[12] Buckley JA, Fishman EK (1998). CT evaluation of small bowel neoplasms: spectrum of disease. Radiographics.

[13] Horton KM, Abrams RA, Fishman EK (2000). Spiral CT of colon cancer: imaging features and role in management. Radiographics.

[14] Gore RM, Balthazar EJ, Ghahremani GG, Miller FH (1996). CT features of ulcerative colitis and Crohn’s disease. AJR Am J Roentgenol.

[15] Balthazar EJ, Gordon R, Hulnick D (1990). Ileocecal tuberculosis: CT and radiologic evaluation AJR. Am J Roentgenol.

[16] Suri S, Gupta S, Suri R (1999). Computed tomography in abdominal tuberculosis. Br J Radiol.

[17] Pereira JM, Sirlin CB, Pinto PS, Jeffrey RB, Stella DL, Casola G (2004). Disproportionate fat stranding: a helpful CT sign in patients with acute abdominal pain. Radiographics.

[18] Padidar AM, Jeffrey RB, Mindelzun RE, Dolph JF (1994). Differentiating sigmoid diverticulitis from carcinoma on CT scans: mesenteric inflammation suggests diverticulitis. AJR Am J Roentgenol.

[19] Jang HJ, Lim HK, Lee SJ, Lee WJ, Kim EY, Kim SH (2000). Acute diverticulitis of the cecum and ascending colon: the value of thin-section helical CT findings in excluding colonic carcinoma. AJR Am J Roentgenol.

[20] Singh AK, Gervais DA, Hahn PF, Rhea J, Mueller PR (2004). CT appearance of acute appendagitis. AJR Am J Roentgenol.

[21] Ahualli J (2005). The target sign: bowel wall. Radiology.

[22] Bartnicke BJ, Balfe DM (1994). CT appearance of intestinal ischemia and intramural hemorrhage. Radiol Clin North Am.

[23] Balthazar EJ, Yen BC, Gordon RB (1999). Ischemic colitis: CT evaluation of 54 cases. Radiology.

[24] Rha SE, Ha HK, Lee SH, Kim JH, Kim JK, Kim JH, Kim PN, Lee MG, Auh YH (2000). CT and MR imaging findings of bowel ischemia from various causes. Radiographics.

[25] Ha HK, Rha SE, Kim AY, Auh YH (2000). CT and MR diagnosis of intestinal ischemia. Semin Ultrasound CT MR.

[26] Thoeni RF, Cello JP (2006). CT imaging of colitis. Radiology.

[27] Roggeveen MJ, Tismenetsky M, Shapiro R (2006). Best cases from AFIP ulcerative colitis. Radiographics.

[28] Philpotts LE, Heiken JP, Westcott MA, Gore RM (1994). Colitis: use of CT findings in differential diagnosis. Radiology.

[29] Merine D, Fishman EK, Jones B (1987). Pseudomembranous colitis: CT evaluation. J Comput Assist Tomogr.

[30] Sivit CJ, Taylor GA, Bulas DI, Kushner DC, Potter BM, Eichelberger MR (1992). Posttraumatic shock in children: CT findings associated with hemodynamic instability. Radiology.

[31] Mirvis SE, Shanmuganathan K, Erb R (1994). Diffuse small-bowel ischemia in hypotensive adults after blunt trauma (shock bowel): CT findings and clinical significance. AJR Am J Roentgenol.

[32] Lane MJ, Katz DS, Mindelzun RE, Jeffrey RB (1997). Spontaneous intramural small bowel hemorrhage: importance of non-contrast CT. Clin Radiol.

[33] Bodily KD, Fletcher JG, Solem CA, Johnson CD, Fidler JL, Barlow JM, Bruesewitz MR, McCollough CH, Sandborn WJ, Loftus EV, Harmsen WS, Crownhart BS (2006). Crohn disease: mural attenuation and thickness at contrast-enhanced CT Enterography-correlation with endoscopic and histologic findings of inflammation. Radiology.

